# Officers' Food, and the Regulation of Meals

**Published:** 1886-10-02

**Authors:** 


### THE EDITOR'S LETTER-BOX.

[Our Correspondents are reminded that prolixity is a great bar to publication, and that brevity of style and conciseness of statement greatly facilitate early publication.]

### OFFICERS' FOOD AND THE REGULATION OF MEALS.

I AM a member of more than one Hospital Committee, and I find that we have a great difficulty to maintain regularity with reference to the hours during which the meals of the resident medical officers and their assistants shall be served and removed. At a provincial hospital, which is under the management of a representative committee, the individual members of which devote much time and attention to the management, serious disagreement has taken place between the lady superintendent and the medical officers on this point. The committee have made rules, which the resident officers seem determined to break. A few weeks ago, at the request of the resident officers, the dinner hour was fixed at seven o'clock, and on three occasions in the following week the house-surgeon did not come to his dinner until after eight, and then created a disturbance because, he said, his food was over-cooked, or badly served, or cold; all of which drawbacks, if true, were but the natural outcome of his own irregularity and defiance of order. Can you, or any of your readers, inform my colleagues and myself whether there is any remedy short of the dismissal of the resident officers at fault, and the strict enforcement of definite rules with regard to the service and removal of meals?

I rejoice to know that your columns will be open to all sides of every question which affects the welfare and the comfort of the resident officers, the patients, and others who reside in, or have part in the management of, hospitals and asylums.

I believe that this question of meals, combined with the kind of food supplied and service of such food, not only in regard to the officers' table, but also as affecting the health and efficiency of the nursing staff, is one of the first importance, and I make no apology, therefore, for raising one aspect of it in your columns. I can assure the resident medical officers, and all who are interested in this question, that the committees of management to which I belong desire, to the utmost of their power, to promote the comfort and happiness of all who labour within the walls of their institutions.

I hope, therefore, that this arrow shot at a venture may awaken a sympathetic echo in the breasts of all who labour in this field of charity, and that those who believe they have cause for complaint or dissatisfaction will give expression to their opinions, and describe their experiences in the columns of THE HOSPITAL, which ought to be the organ of the governors, the officials, and the patients, as well as of the public, in the best sense and meaning of that word.

It would be interesting to have a *menu* or diet-table, if any such exist, for the regulation and service of the meals of the officers and nurses, or others, over a given period of time. I have often noticed an absence of variety in the food-supply, and I fear that there may be a too close adherence to the regulation "leg of mutton and trimmins," if a due regard be had to the health and well-being, and therefore to the best services of the officers of all grades, who labour in the cause of suffering humanity, in the public medical institutions of this country.

A HOSPITAL MANAGER AND TRUSTEE.

*** We shall be glad to have the views of all who may be interested in the food-supply and regulation of meals at public institutions. If it be possible to carry out the suggestion of our correspondent by sending the *menu* or diet-table which regulates the supply of meals at any hospital or asylum, we will do our best to find room for it, or, at any rate, to make a selection from those sent. We have been favoured with the following *menu*, which was successfully introduced at the Seamen's Hospital some years ago, and which gave satisfaction to the nurses by promoting their comfort and health in every way.—ED. T. H.

LIST OF DINNERS.

MONDAY.—Boiled Mutton, Carrots, and Turnips.
TUESDAY.—Boiled Salt Beef with Suet Pudding.
WEDNESDAY.—Roast Beef and Cabbage.
THURSDAY.—Roast Mutton or Veal.
FRIDAY.—Boiled Mutton or Fish, with light Pudding.
SATURDAY.—Liver and Bacon, Irish Stew, or any made dish, with Milk Puddings.
SUNDAY.—Roast Beef or Pork, with Tart or Pudding.

